# Late-Onset Contrast-Induced Encephalopathy After Stent-Assisted Coiling: A Case Report and Review of the Literature

**DOI:** 10.3390/brainsci16080785

**Published:** 2026-07-26

**Authors:** Gaby Bühler, Michael Diepers, Gerrit A. Schubert, Lukas Andereggen, Serge Marbacher

**Affiliations:** 1 Department of Neurosurgery, Kantonsspital Aarau, CH-5001 Aarau, Switzerland; gaby.buehler@ksa.ch (G.B.);; 2 Institute of Neuroradiology, Kantonsspital Aarau, CH-5001 Aarau, Switzerland; 3 Department of Neurosurgery, RWTH Aachen University, D-52062 Aachen, Germany; 4 Department of Neurosurgery, Kantonsspital Aarau, University of Bern, CH-5001 Aarau, Switzerland

**Keywords:** contrast-induced encephalopathy, angiography, contrast, neurotoxicity, complication

## Abstract

**Highlights:**

**Main findings:**

- CIE can present with delayed symptom onset beyond 24 h following contrast administration.
- Corticosteroid therapy may contribute to symptom improvement by reducing inflammatory processes.

**Implications of the findings:**

- Late-onset CIE should be considered because symptoms may emerge several days after contrast administration.
- Extended postprocedural observation may be beneficial in selected patients with risk factors for CIE.
- Standardized treatment protocols for CIE require further investigation.

**Abstract:**

Introduction: Contrast-induced encephalopathy (CIE) is a rare condition with a broad spectrum of neurological symptoms. With the increasing use of endovascular techniques, the disease is becoming ever more important. To date, recent studies have primarily described patients with early-onset CIE, in which symptoms appear shortly after the intervention. This case report represents one of the few reported cases of late-onset CIE. Case description: A 53-year-old woman with a re-recurrent anterior communicating artery aneurysm with coil impaction and a history of two aneurysmal subarachnoid hemorrhages underwent stent-assisted re-recoiling without incident. Some 31 h after the procedure, she experienced a generalized tonic–clonic seizure. Postprocedural magnetic resonance imaging (MRI) earlier that day had shown slight cortical swelling and mild leptomeningeal effusion in the left frontal lobe, without any other abnormalities. Computerized tomography (CT) imaging afterwards ruled out intracranial hemorrhage. Following an otherwise uneventful clinical course, the patient developed impaired vigilance, right-sided leg weakness, and fine-motor impairment of the hand on the sixth day after the intervention. Subsequent MRI demonstrated patent intracranial arteries and no evidence of ischemia. However, the imaging findings were consistent with CIE, showing characteristic cortical swelling and signal alterations in the sulcal subarachnoid space. In addition, multiple cerebral microbleeds were identified. After the initiation of dexamethasone therapy, the patient exhibited prompt neurological improvement, with full neurological recovery at the three-month follow-up. Conclusions: This case appears to be one of the rare documented occurrences of late-onset CIE, highlighting the importance of postinterventional neurological monitoring. Furthermore, it suggests the potential role for high-dose corticosteroid therapy.

## 1. Introduction

Contrast-induced encephalopathy (CIE) is a rare but potentially serious neurological complication that can occur after the intra-arterial application of iodinated contrast agents. Recent studies, including a large multicenter cohort study involving 5126 patients, report an incidence of CIE ranging from 0.3% to 1%. There are reasonable assumptions, though, that the syndrome is markedly underdiagnosed. A current study reported an incidence of almost 5% [1,2,3,4,5,6,7].

The pathogenesis of CIE is assumed to be related to a disruption of the blood–brain barrier (BBB) due to osmotic effects and direct neurotoxicity of the contrast agent. Furthermore, the institutional case series and literature review by Maclean et al. identified that procedure length, the administered volume of contrast medium, severe intraoperative hypertension, and the use of mechanical devices resulting in iatrogenic endothelial injury are risk factors for the development of CIE [3]. The symptoms of CIE vary widely and may include headache, loss of consciousness, seizures, and focal neurological impairments such as hemiparesis, blindness, and aphasia. Typically, these manifestations arise within a few hours after exposure to iodinated contrast agents [2,3,4,5,6,7,8].

While acute cases are well-documented, late-onset CIE, particularly following cerebral angiography, remains poorly understood and rarely reported. However, contrast-induced encephalopathy (CIE) has not been limited to cerebral angiography; it has also been observed in various other angiographic procedures, particularly coronary angiography [9]. Imaging techniques such as computerized tomography imaging (CT) and magnetic resonance imaging (MRI) are crucial to confirming the diagnosis and distinguishing CIE from other neurological conditions.

In this case report, we present a patient who developed late-onset CIE after the angiographic stent-assisted recoiling of an aneurysm. We aim to highlight the uncommon presentation and discuss the diagnostic and therapeutic challenges associated with its management. This manuscript was prepared in accordance with the CARE (CAse REport) reporting guidelines [10], Supplementary Material.

## 2. Case Report

A 53-year-old female patient had a history of an aneurysmal subarachnoid hemorrhage (SAH) of a broad-based anterior communicating artery (ACoA) aneurysm in 2010 (Hunt and Hess grade 1, Fisher grade 1). In consideration of the aneurysm’s morphology and the patient’s age, it was decided to perform emergency clipping. Apart from a minor inferior nasal quadrantanopia, the patient achieved full neurological recovery.

In 2024, the patient suffered another severe aneurysmal SAH (Hunt and Hess grade 3, Fisher grade IV), due to a recurrence of the clipped ACoA aneurysm. Regarding the irregular configuration with trilobulated recurrence and micro-lobulations, the decision was made to pursue endovascular treatment in the acute phase of SAH. Consequently, conventional coiling was performed with a small basal residual. Again, the patient recovered well from the recurrent SAH but showed mild cognitive deficits and frontal brain involvement with a modified Rankin Scale of 1 (mRS).

Subsequently, in the six-month control angiography, a new recurrence at the aneurysmal neck with coil impaction was detected, requiring re-intervention. At that time, the patient had preserved renal function with an estimated glomerular filtration rate >90 mL/min/1.73 m^2^, and serum creatinine within the normal range at 56 µmol/L. The access catheter for the procedure had been brought up to the distal internal carotid artery (ICA) and, for stent deployment, up to the left anterior cerebral artery (ACA) A1 segment. Because there was an aplasia of the A1-segment of the contralateral ACA, the ACA and ICA runs always included both ACA territories. A total volume of 367 milliliters (mL) of iopamidol, a non-ionic low-osmolar contrast agent, was administered, including 275 mL into the left ICA and 30 mL into the left ACA A1 segment. The procedure was successful, without incident; the patient woke up on time and had no new deficits.

**Figure 1:**
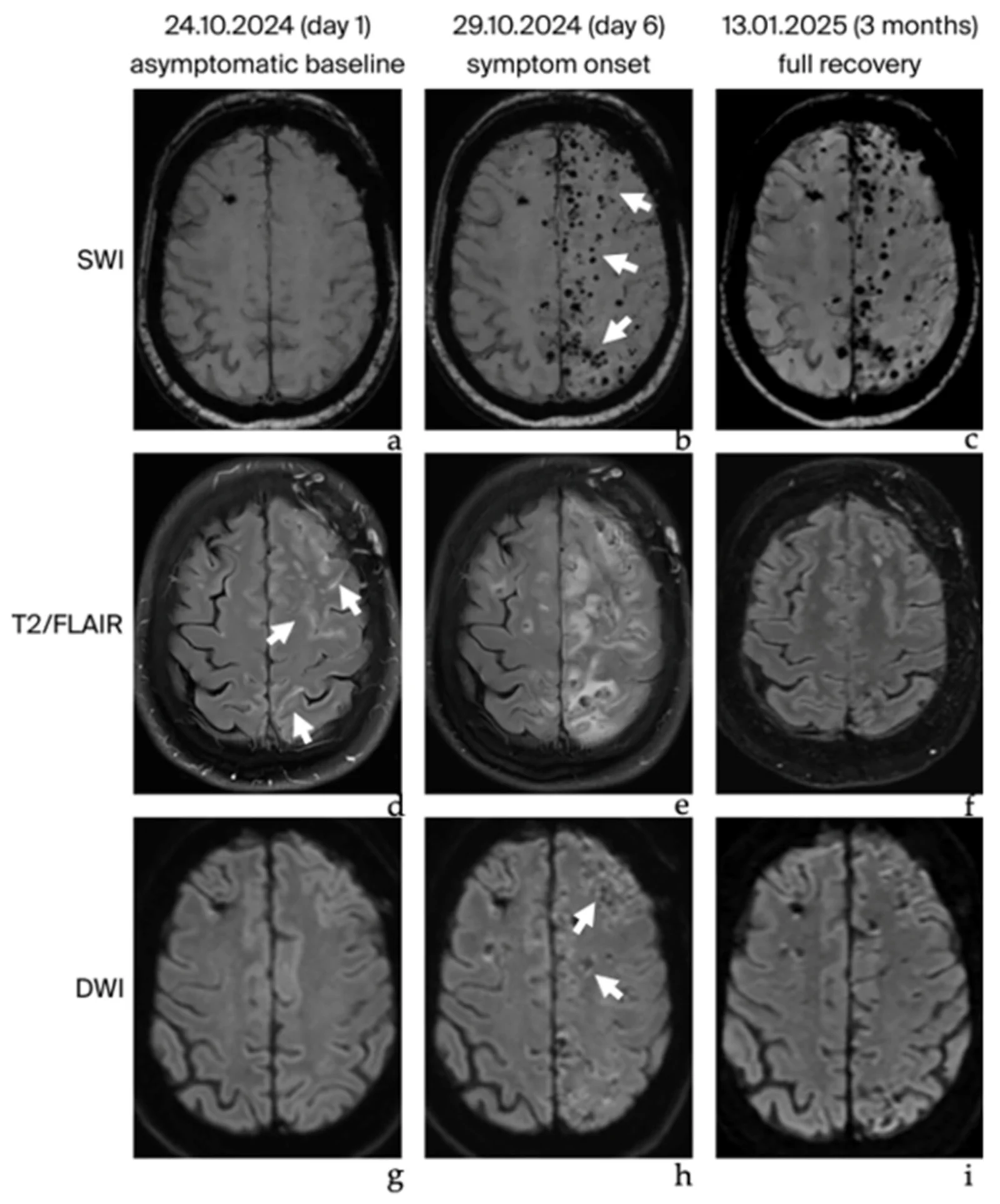
Timeline of brain magnetic resonance imaging findings of late-onset CIE. (**a**–**c**): Susceptibility weighted imaging (SWI) demonstrating multiple cerebral microbleeds at symptom onset (Image (**b**)—arrows). (**d**–**f**): T2-weighted fluid attenuated inversion recovery (FLAIR) imaging showing mild leptomeningeal effusion on day 1 (Image (**d**)—arrows), progression at symptom onset (Image (**e**)), and complete resolution at the 3-month follow-up (Image (**f**)). (**g**–**i**): Diffusion-weighted imaging (DWI) demonstrating patchy cortical and subcortical signal abnormalities at symptom onset (Image (**h**)—arrows) with regression at the 3-month follow-up (Image (**i**)).

The day after intervention, a regular MRI was performed, which showed mild T2-weighted fluid-attenuated inversion recovery (T2-FLAIR) hyperintensities on the left frontal lobe (Figure 1), without susceptibility-weighted imaging (SWI) irregularities. On the same day, 31 h after the intervention, she had a tonic–clonic seizure. A CT scan was performed, but without any new abnormalities and regular CT angiography. In the following days, the patient showed no neurological deficits until a new right hemiparesis and cognitive impairments occurred at the sixth day post-treatment. The emergency MRI revealed multiple cerebral microbleeds in the SWI and a marked increase in T2-FLAIR hyperintensities in the left hemisphere (Figure 1). Thereafter, short-term glucocorticoid therapy was administered for three days. Specifically, 8 milligrams (mg) of dexamethasone was administered on the day of diagnosis, followed by 12 mg orally once daily for the next two days. Subsequently, the patient showed a mild regression in symptoms; however, her mobility remained severely impaired, requiring assistance. This was accompanied by marked fine motor weakness on the right side (mRS 4). Therefore, neurological rehabilitation was initiated.

Finally, at the three- and six-month follow-up visits, the patient demonstrated complete clinical remission of symptoms. Her functional status remained at her pre-intervention baseline, with a mRS of 1 due to mild cognitive deficits. MRI revealed persistent SWI abnormalities, while T2-FLAIR hyperintensities had completely resolved.

## 3. Discussion

Numerous case reports and cohort studies have documented the occurrence of early-onset CIE. Nonetheless, emerging clinical observations suggest that CIE may not be limited to early presentations, complicating diagnosis and raising concerns regarding standard postprocedural monitoring protocols. Due to the limited number of published cases, late manifestations are probably underrecognized and frequently misattributed to other neurological conditions (Table 1).

In this case report, we present one of the rare documented cases of late-onset CIE. In the case described, the initial clinical course after the intervention was unremarkable. A routine MRI control scan performed the following day revealed mild nonspecific local T2-FLAIR changes on the left frontal lobe. Later the same day, and without any documented prodromal symptoms, the patient had a generalized tonic–clonic seizure. A subsequent CT scan showed only the previously known mild neuroradiological abnormalities, including slight cortical swelling and very subtle leptomeningeal effusions, without evidence of intracranial hemorrhage. Notably, there was no radiological indication of contrast agent extravasation, which is typically suggestive of CIE. As a result, the etiology of the seizure remained unclear at that time. Although CIE was discussed, it was considered unlikely due to the absence of characteristic findings. Nevertheless, diagnostic work-up did not identify an alternative diagnosis. Differential diagnoses, such as ischemic stroke, posterior reversible encephalopathy syndrome (PRES), infection, or other causes of encephalopathy, were excluded by laboratory tests and neuroimaging.

Six days after intervention, however, the patient developed hemiplegia and decreased alertness. These new clinical symptoms coincided with additional imaging findings, including multiple newly developed cerebral microbleeds, a slight increase in cortical swelling, and new leptomeningeal effusions on MRI (Figure 1). Based on the progression of radiological abnormalities and the lack of alternative identifiable causes, the diagnosis of CIE was subsequently established. Although the initial MRT und CT scans were unremarkable, several studies have shown that imaging findings can vary widely, ranging from no abnormalities to the typical cortical contrast staining with T2-FLAIR hyperintensity in cortical and subcortical regions, cerebral edema, sulcal effacement, and subarachnoid contrast enhancement. The literature consistently emphasizes that unremarkable imaging findings do not rule out a diagnosis of CIE. Due to the lack of standardized diagnostic criteria, Mariajoseph et al. proposed the first consensus-based diagnostic criteria for CIE in 2025. These criteria comprise 14 diagnostic items, including absolute exclusion criteria, such as symptom onset more than 24 h after contrast administration. Accordingly, our case would not meet these proposed diagnostic criteria, as the seizure occurred more than 24 h after contrast administration [3,11,12,13].

However, the cerebral microbleeds detected on MRI performed 7 days after the intervention may represent a radiological manifestation of contrast-induced BBB dysfunction. Maclean et al. suggested that BBB disruption may be related to the chemotoxic effects of contrast agents on the cerebral microvasculature, including reduced expression of endothelial tight junction proteins, thereby increasing vascular permeability [3]. Furthermore, they proposed that pre-existing microvascular disease, such as cerebral small vessel disease, hypertension, diabetes mellitus, chronic kidney disease, smoking, advanced age, and prior cerebrovascular disease, may be relevant risk factors for BBB disruption and the development of CIE. Whether repeated angiographic procedures with contrast agent exposure may also contribute to pre-existing microvascular vulnerability remains unclear and warrants further investigation. This aspect may be of relevance in our patient, who had repeated contrast agent exposure. She underwent three angiographic procedures within one year prior to the event and had a prolonged recovery course lasting several weeks, contrasting with most reported cases to date, in which symptoms typically resolved within hours to a few days [2,3,5,6,7,8,9,11,12,14,15,16].

As mentioned in the previous section, the amount of contrast agent administered has been suggested to be a relevant risk factor in the development of CIE. Our patient received a total of 367 mL of contrast agent, 305 mL of which was administered into intradural arteries. This amount of contrast agent seems to be within the range reported for neurointerventional procedures in previous studies. However, current evidence does not support a defined dose threshold, and CIE has been reported across a broad range of contrast volumes [3,5,6]. Furthermore, Fuga et al. identified intradural catheter placement and previously treated aneurysm as independent predictors of CIE. In addition, Vigan et al. proposed that recent SAH may impair BBB integrity through endothelial injury. In our patient, who had experienced recurrent SAH, pre-existing BBB dysfunction may therefore have constituted an additional predisposing factor for CIE, despite the absence of persistent neurological deficits [14].

Despite the lack of standardized treatment recommendations, corticosteroids have been proposed as a potential therapeutic option [9]. Their use is largely based on the proposed pathophysiology of BBB disruption, vasogenic edema, and secondary neuroinflammatory responses [17]. Glucocorticoids may stabilize the BBB, reduce endothelial permeability, and attenuate inflammatory cascades, thereby limiting further neuronal injury. However, the available evidence is limited to case reports and small case series. Mariajoseph et al. reported that corticosteroid administration was significantly associated with symptom resolution within 72 h (OR 4.51, 95% CI 1.19–17.85, *p* = 0.022). Moreover, as most patients recover with supportive management alone, the specific contribution of corticosteroids remains inconclusive. Nevertheless, in our patient, neurological improvement was observed shortly after corticosteroid therapy was initiated. Whether this association reflects a true treatment effect or the natural course of CIE cannot be determined.

In this case, whether the onset of the syndrome occurred on the evening following the intervention or five days later remains controversial. However, it is obvious that new neurological deficits occurred in a delayed fashion and that the patient’s recovery extended over several weeks. This unexpected clinical course highlights the need for increased clinical vigilance and extended monitoring in patients at risk of CIE, especially those with initially normal imaging findings and evolving neurological symptoms. Furthermore, clinicians should pay particular attention to patients with predisposing factors. Therefore, extending the observation period in selected cases may help in early identification and appropriate management of late-onset complications.

**Table 1:** Literature survey of early-onset CIE.

Author	Year	Indication (*n*)	Contrast Agent and Volume (mL Median)	Treatment (*n*)	No. of Cases (*n*)	Symptom Onset (Median/Latest Onset)	Clinical Outcome (*n*)
Maclean et al. [3]	2025	EVT (*4*) Neurointervention for aneurysm (*2*)	Non-ionic, low osmolality (270 mL)	Corticosteroids Supportive	*6*	45 min/165 min	Complete recovery in most patients
Mariajoseph et al. [9]	2024	Cerebral DSA ± intervention (*33*) Coronary angiography ± intervention (*31*) Other (*9*)	Non-ionic, iso and low osmolality (150 mL)	Corticosteroids Supportive Mannitol	*73*	1 h/27 h	Complete recovery (*66*) Deficits/Death (*11*)
Stebner et al. [11]	2024	EVT (*2*) Neurointervention for aneurysm (*2*) Cerebral DSA (*1*) Coronary angiography (*1*) Other (*1*)	Non-ionic, low osmolality (range 100–300 mL)	Corticosteroids Supportive Hemodialysis	*7*	n.A./7 h	Complete recovery in most patients, 1 death
Mariajoseph et al. [2]	2024	Neurointervention for aneurysm (*4*)	Non-ionic, low osmolality (200 mL)	Corticosteroids (*3*) Supportive (*4*) Mannitol (*1*)	*4*	n.A./10 h	Complete recovery (*2*) Residual deficits (*2*)
Fuga et al. [16]	2023	Cerebral neurointervention (*14*)	Non-ionic, low osmolality (173 mL)	Supportive Hemodialysis	*14*	1 h/n.A.	Complete recovery in most patients
Jankovic et al. [4]	2023	Neurointervention for aneurysm (*1*) Cerebral DSA (*1*)	Non-ionic, low osmolality (60 mL)	Corticosteroids Supportive	*2*	16.5 d/4 w	Residual numbness
Allison et al. [12]	2021	Neurointervention for aneurysm (*2*) For arteriovenous malformation (*1*) For carotid vessel dysplasia (*1*)	Non-ionic iso and low osmolality (118 mL)	Corticosteroids Supportive Mannitol	*4*	3.5 h/6 h	Complete recovery in most patients
Vigano et al. [14]	2021	Cerebral DSA (*1*)	Non-ionic, low osmolality (70 mL)	Corticosteroids Sodium-chloride	*1*	During intervention	Complete recovery
Chu et al. [5]	2020	EVT (*7*)	Non-ionic, low osmolality (32 mL)	Supportive	*7*	n.A./24 h	mRS 4 (median)
Harada et al. [7]	2020	Coronary angiography (*1*)	Non-Ionic, low osmolality (210 mL)	Supportive	*1*	Immediately after intervention	Complete recovery
Leong et al. [15]	2012	Neurointervention for aneurysm (*1*)	Non-ionic, low osmolality (220 mL)	Corticosteroids Mannitol	*1*	Immediately after intervention	mRS 2-residual spasticity

EVT: endovascular thrombectomy; DSA: digital subtraction angiography; n.A.: not available; mL: milliliters, min: minutes; h: hours; d: days; w: weeks; mRS: modified Rankin Scale.

## 4. Conclusions

This case description highlights the increasing evidence of late-onset CIE following endovascular procedures and underlines the importance of increased clinical vigilance with potential extended monitoring to improve safety in endovascular intervention and the need for comprehensive risk assessment. Corticosteroid therapy showed potential benefits in mitigating inflammatory processes. Nevertheless, further research is needed to establish a standardized treatment protocol.

## Data Availability

Data available on request due to privacy restrictions.

## Supplementary Materials

The following supporting information can be downloaded at: https://www.mdpi.com/article/10.3390/brainsci16080785/s1, CARE reporting guidelines.
